# Changes in patellar morphology following surgical correction of recurrent patellar dislocation in children

**DOI:** 10.1186/s13018-021-02779-7

**Published:** 2021-10-16

**Authors:** Weifeng Li, Qian Wang, Hui Li, Shunyi Wang

**Affiliations:** 1 Department of Orthopaedic Surgery, The First Central Hospital of Baoding, No. 320 Changcheng Street, Baoding, 071000 Hebei People’s Republic of China; 2 The First Department of Operating Room, The First Central Hospital of Baoding, No. 320 Changcheng Street, Baoding, 071000 Hebei People’s Republic of China

**Keywords:** Knee, Recurrent patellar dislocation, Children, Patella, Morphology

## Abstract

**Background:**

The aim of this study was to evaluate patellar morphological changes following surgical correction of recurrent patellar dislocation in children.

**Methods:**

A total of 35 immature children aged 5 to 10 years who suffered from bilateral recurrent patellar dislocation associated with abnormal patella morphology were enrolled in this study. The knees with the most frequent dislocations (treated with medial patellar retinacular plasty) were selected as the study group (SG), and those undergoing conservative treatment for the contralateral knee were selected as the control group (CG). Computed tomography (CT) scans were performed on all children preoperatively and at the last follow-up to evaluate morphological characteristics of the patella.

**Results:**

All the radiological parameters of the patella showed no significant difference between the two groups preoperatively. At the last follow-up for CT scans, no significant differences were found for the relative patellar width (SG, 54.61%; CG, 52.87%; *P* = 0.086) and the relative patellar thickness (SG, 26.07%; CG, 25.02%; *P* = 0.243). The radiological parameters including Wiberg angle (SG, 136.25°; CG, 122.65°; *P* < 0.001), modified Wiberg index (SG, 1.23; CG, 2.65; *P* < 0.001), and lateral patellar facet angle (SG, 23.35°; CG, 15.26°; *P* < 0.001) showed statistical differences between the two groups.

**Conclusions:**

The patellar morphology can be improved by early surgical correction in children with recurrent patellar dislocation. Therefore, early intervention is of great importance for children diagnosed with recurrent patellar dislocation.

## Introduction

The patella plays an important role in human activities. Patellar dislocation is a common medical condition in clinical practice, which has the highest incidence among children and adolescents, reaching approximately 147.7 per 100.000 person-years [1]. Lewallen et al. [2, 3] reported that the redislocation rates of up to 71% among immature patients. Jaquith and Parikh found that adolescents with open physis had more than twice the risk of recurrent patellar dislocation compared to patients with closed physis [4, 5]. Dejour et al. [6] showed that 96% of patients with patellar dislocation had trochlear dysplasia. Servien and Li found that patients with trochlear dysplasia had a patella of smaller width, thinner thickness, more flattened articular facet and increased Wiberg index [7, 8]. Jaquith and Parikh reported a particularly evident fact that the simultaneous presence of a patellar and trochlear dysplasia increased the risk of redislocation to more than 70% [4, 5]. Fu et al. [9] have recently showed that early relocation of the patella could prevent the development of trochlear dysplasia in children. However, to our knowledge, no authors have described the potential effect of patellar correction on the development of the patella in children. The aim of the present study was to compare the changes in patellar morphology following surgical correction for recurrent patellar dislocation in children.

## Material and methods

This study was approved by the Ethics Committee of our hospital, and all patients provided informed consent.

All of the children had a history of bilateral recurrence and were diagnosed as disruption of the normal position of patella within the trochlea groove because of multiple traumatic episode [10]. These two independent senior orthopedic observer defined the bilateral recurrent patella dislocation on knees. The exclusion criterion was a history of prior surgery, ligament injury or cartilage damage of greater than grade II [11].

We enrolled 35 patients (23 females and 12 males, with a mean age of 7.8 years [range, 5–10 years]) who had bilateral recurrent patellar dislocation associated with abnormal patella morphology that was diagnosed according to Askenberger M [12]. The knee in the study group (SG) was treated with medial patellar retinacular plasty. The patients whose contralateral knee was treated conservatively were selected as the control group. They were followed up from February 2008 to December 2014, with a mean of 78.6 months (range, 62–106 months). The 35 immature patients [mean age, 7.8 years (range, 5–10 years)] with recurrent patellar dislocation underwent surgical or conservative treatment in the study. Each patient had a tibial tuberosity–trochlear groove distance (TT–TG), congruence angle (CA) and patellar tilt angle ( PTA) examination on CT scans to assess the alignment of the patello-femoral joint preoperatively and at the last follow-up [13, 14]. According to CT images of the knee joint, the patellar morphology was analyzed on particular axial views. Furthermore, function of the knee joint was evaluated by using the apprehension test, Kujala score and Crosby and Insall grading system [15–17]. All controls consulted the orthopedic surgeon for a complaint, such as soft cartilage injury.

### Surgical procedures

As for surgical techniques in children, the risk of growth plate injury and open epiphysis should be considered [18–20]. Therefore, numerous bony procedures were contraindicatory, and soft-tissue procedures were designed according to the anatomy. The surgical technique employed had been previously described in the study [9]. All surgeries were performed by two senior surgeons. All patients underwent general anesthesia and arthroscopic assessment by standard procedures to deal with intra-articular lesions before medial patellar retinacular plasty treatment. A medial shift force of the patella of less than one quarter the width of the patella indicated the overtension of lateral retinaculum, and the lateral retinacular release was performed in these cases [21]. In SG, eight patients received arthroscopic lateral retinacular release. After the diagnostic arthroscopy, the subsequent procedure of medial patellar retinaculum (MPR) plasty was enumerated. First, a 3-cm incision was created on the medial margin of the patella. The vastus medialis oblique (VMO) and the MPR were exposed and dissected. Next, the position and tension of two structures were divided and adjusted appropriately. The patellar trajectory was dynamically observed under arthroscopy. Then, the MPR was sutured on the medial margin of the patella, the VMO was sutured on the edge of the MPR with PDS-II (polydioxanone) synthetic absorbable suture (Ethicon LLC, USA) to restore the superior-oblique bundle of the MPR. Also, the overlapped tissues were sutured together using PDS-II. Finally, the incision was irrigated and sutured in layers.

### Rehabilitation

The postoperative rehabilitation process included the use of knee immobilizers for 2 weeks [22–24]. For the first few days, to minimize the risk of arthrofibrosis, continuous passive or active motion should be applied postoperation. Full weight bearing and athletic exercises should be performed at 4–10 weeks after operation [25].

### Conservative management

Conservative treatment played a primary role in each stage of the CG in patients [26], which was initiated along with the surgery on the contralateral knee. This immobilization in a brace and physiotherapy should be included [27–29]. Physiotherapy (including straight leg raise, isometric quadriceps, wall slide exercise and closed and open chain rehabilitation) should be exercised in each patient undergoing conservative treatment, which helped to control lateral instability in the tight lateral retinaculum [27–32]. Patients should be instructed to perform the above exercises 7 times each, 3 times daily. Bitar et al. [30] stated that physiotherapy was a key factor to improve quadriceps strength. McConnell showed good results in conservative patients using physiotherapy and the brace taping technique to modify patellar tracking for 12 months [29]. Therefore, the study involved treatment with physiotherapy, and the brace taping technique was undertaken in these patients every day for more than 12 months.

### Clinical assessments

**Fig. 1:**
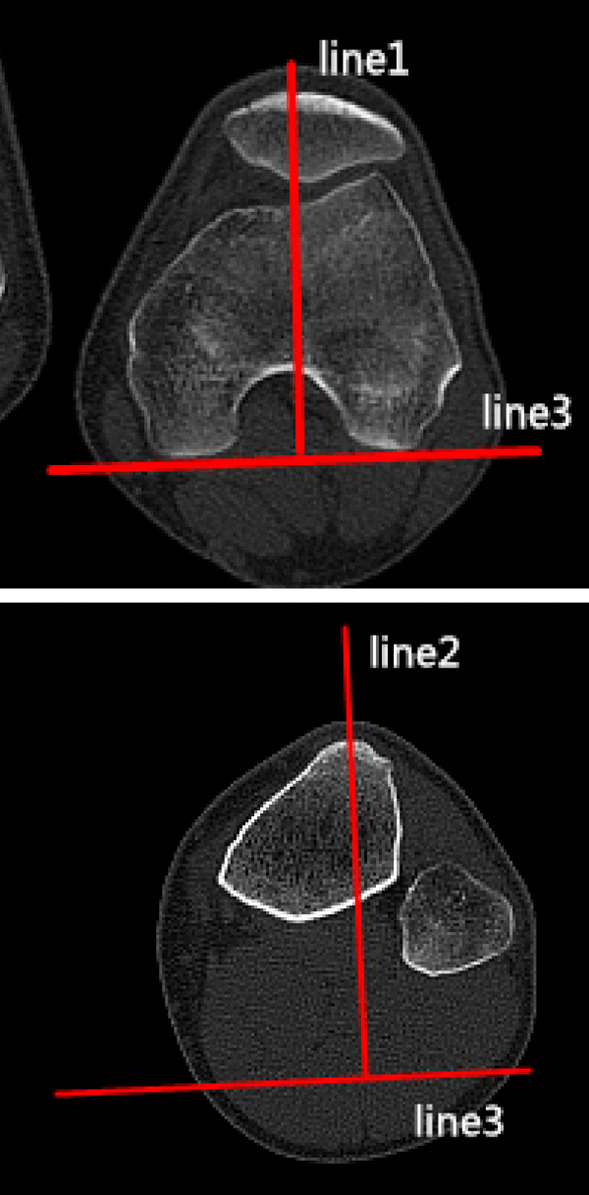
Tibial tuberosity–trochlear groove distance (TT–TG) The line 1 was drawn through the bottom of the trochlear groove, while the line 2 was drawn through the middle point of the tibial tubercle. Line 1 and line 2 were perpendicular to the tangent line of the dorsal femoral condylar line (line 3). The TT–TG distance was the distance between the two parallel lines

**Fig. 2:**
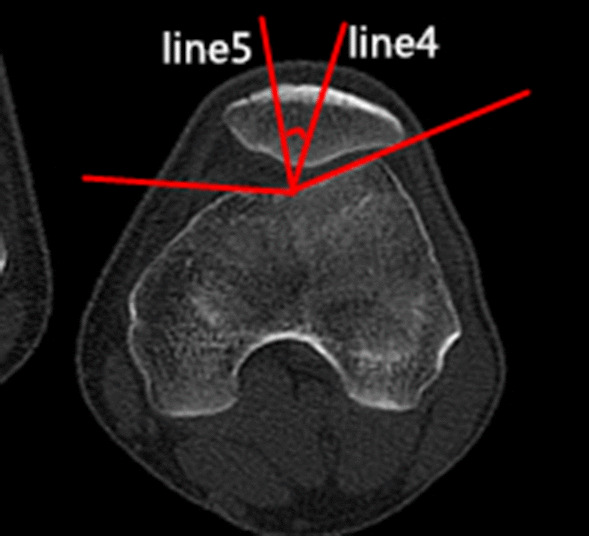
The congruence angle (CA) is the angle with the line drawn through the lower pole of the patella and the deepest point of the chute (line 4) to the line on the side of the bisector defines the tackle angle (line 5)

**Fig. 3:**
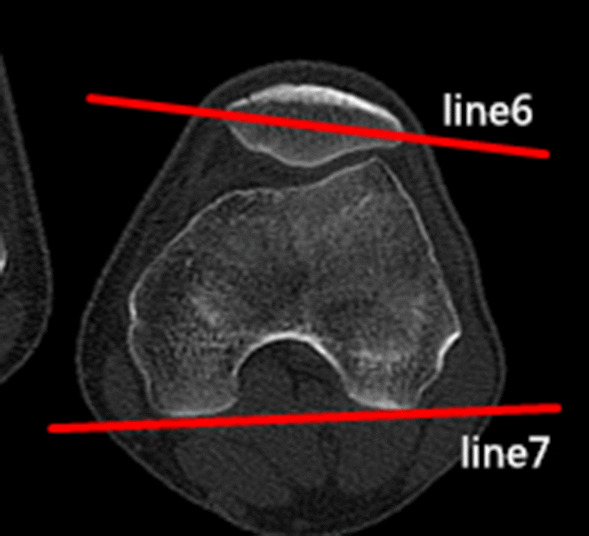
The patellar tilt angle (PTA) is the angle between the extension line of the maximum transverse diameter of the patella (line 6) and the tangent to the posterior condyles (line 7)

**Fig. 4:**
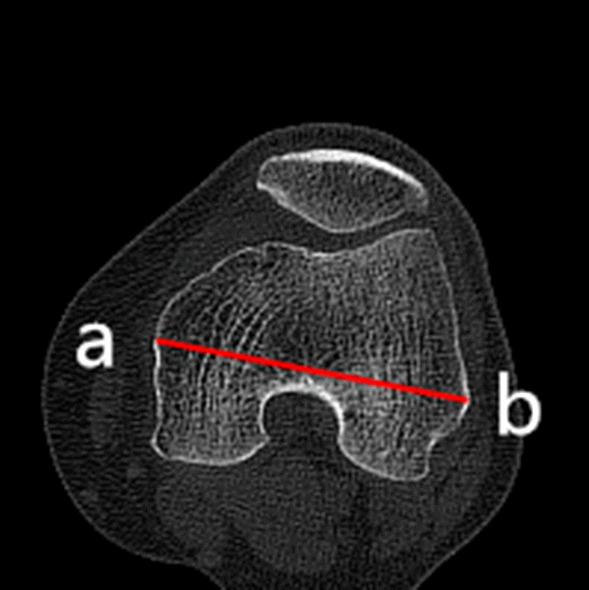
The medial–lateral width (MLW) is the length between the medial (a) and lateral edge (b) of the epicondyle

**Fig. 5:**
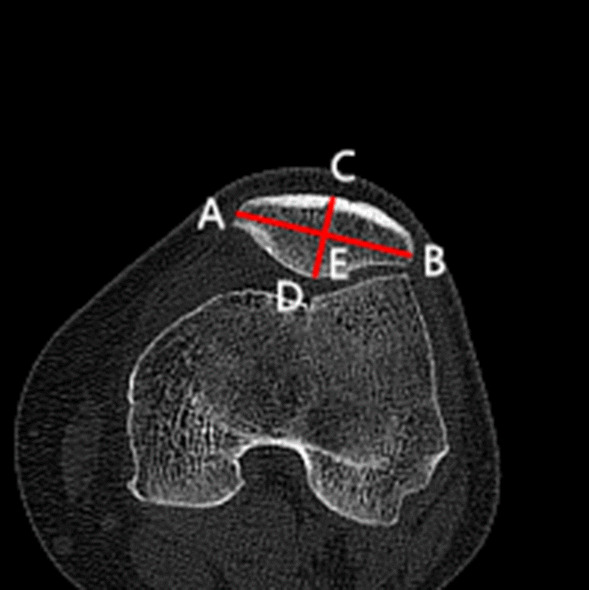
The patellar width (PW) is the length between the medial (**A**) and lateral edge (**B**) of the patella. The patellar thickness (PT) is the length between the patellar front polar (**C**) and back polar (**D**). The modified Wiberg index is defined as the ratio of the transverse length of the lateral patellar facet (AE) to the medial patellar facet (BE)

**Fig. 6:**
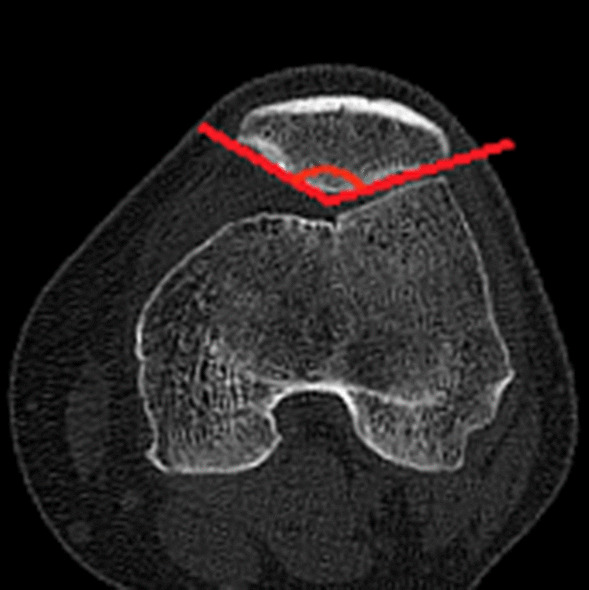
The Wiberg angle is the angle formed by the medial and the lateral patellar facet tangent

**Fig. 7:**
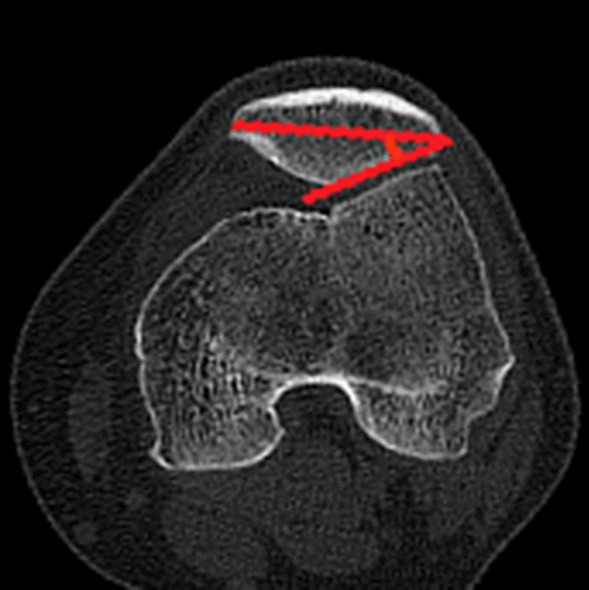
The lateral patellar facet angle is the angle formed by the patellar transverse axis and the lateral patellar facet tangent

The diagnosis of recurrent patellar dislocation was confirmed by the CT of the knee joint and patellar apprehension test. CT examinations were performed to assess the morphological characteristics of the patella in the supine position preoperatively and postoperatively (at the last follow-up). CT scans were performed using a Sante DICOM Viewer Free (64-bit) version (Santesoft, Inc. Athens, Greece) to 0.01° for angles and 0.01 mm for distance, and all parameters were assessed utilizing the axial views using 1-mm slices. The relative patellar width, the relative patellar thickness, the Wiberg angle, the modified Wiberg index, and lateral patellar facet angle were measured on the particular axial CT scan [8, 33]. The methods applied to the assessment of alignment of the patello-femoral joint and morphology are summarized in Table 1 and Figs. 1, 2, 3, 4, 5, 6 and 7. In order to minimize error, all measurements were taken by two blinded authors (experienced orthopedic surgeons) using the RadiAnt-DICOM software (Medixant Ltd, Poznan, Poland).

**Table 1:** Description of measurements

*Patella morphological characteristics*	
Tibial tuberosity–trochlear groove distance (TT–TG)	Line 1 was drawn through the bottom of the trochlear groove, and line 2 was drawn through the middle point of the tibial tubercle. Line 1 and line 2 were perpendicular to the tangent line of the dorsal femoral condylar line (line 3). The TT–TG distance was the distance between the two parallel lines (Fig. 1)
Congruence angle (CA)	It was defined as the angle with the line drawn through the lower pole of the patella and the deepest point of the chute (line 4) to the line on the side of the bisector defined the tackle angle (line 5) (Fig. 2)
Patellar tilt angle (PTA)	It was defined as the angle between the extension line of the maximum transverse diameter of the patella (line 6) and the tangent to the posterior condyles (line 7) (Fig. 3)
Medial–lateral width (MLW)	It was defined as the length between the medial (a) and lateral edge (b) of the epicondyle (Fig. 4)
Patellar width (PW)	It was defined as the length between the medial (A) and lateral edge (B) of the patella in the slide with the widest patellar diameter (Fig. 5)
Patellar thickness (PT)	It was defined as the length between the patellar front polar (C) and back polar (D) (Fig. 5)
Modified Wiberg index (AE/BE)	It was measured as the ratio of the transverse length of the lateral patellar facet (AE) to the medial patellar facet(BE) (Fig. 5)
Wiberg angle	The angle between the slopes of the medial and lateral patella (Fig. 6)
Relative patellar width (PW/MLW)	The ratio of length of patellar width to medial–lateral epicondyle width
Relative patellar thickness (PT/MLW)	The ratio of length of patellar thickness to medial–lateral epicondyle width
Lateral patellar facet angle	The angle between the patellar transverse axis (AB) and the lateral patellar facet tangent (Fig. 7)

The mean follow-up was 78.6 months (range 62–106 months), and all assessments were performed preoperatively and at the last follow-up after surgery. The apprehension test was used to evaluate patellar stability, and the Kujala score was administered to evaluate knee function. The Crosby and Insall grading system was used to assess the results of the treatment, which were classified into four categories (excellent, good, fair, and poor). Finally, further episodes of patellar instability were recorded.

### Statistical analysis

Statistical analysis was performed using SPSS software (version 22.0; SPSS, IL, USA). Descriptive statistics were used to evaluate the distribution of continuous data. The normality of numerical data was assessed by the Kolmogorov–Smirnov test, and the homogeneity of the data was assessed by Levene’s test. After establishing data normality, student's paired *t*-test was used to evaluate the differences between the two groups. *P* < 0.05 were defined as the threshold for statistical significance.

## Results

The inter-and intra-observer correlation coefficients were high between measurements (Table 2).

**Table 2:** Intra-observer and inter-observer agreement of geometric measurements with 95% confidence intervals

Measurement	Intraobserver		Interobserver	
	ICC	95% CI	ICC	95% CI
Pre-SG TTTG	0.762	0.665–0.885	0.716	0.603–0.890
Pre-SG CA	0.846	0.781–0.943	0.852	0.790–0.946
Pre-SG PTA	0.840	0.764–0.868	0.796	0.727–0.870
Pre-SG RPW	0.786	0.673–0.935	0.724	0.563–0.869
Pre-SG RPT	0.806	0.720–0.932	0.752	0.595–0.912
Pre-SG WA	0.843	0.636–0.928	0.786	0.527–0.904
Pre-SG MWI	0.882	0.757–0.916	0.757	0.478–0.907
Pre-SG LPFA	0.973	0.943–0.987	0.962	0.921–0.987
Post-SG TTTG	0.755	0.702–0.820	0.731	0.673–0.886
Post-SG CA	0.852	0.783–0.934	0.844	0.756–0.927
Post-SG PTA	0.833	0.782–0.902	0.818	0.725–0.918
Post-SG RPW	0.795	0.802–0.924	0.738	0.773–0.906
Post-SG RPT	0.762	0.773–0.905	0.746	0.736–0.918
Post-SG WA	0.883	0.753–0.960	0.858	0.825–0.916
Post-SG MWI	0.933	0.913–0.965	0.879	0.840–0.954
Post-SG LPFA	0.976	0.958–0.985	0.916	0.827–0.969
Pre-CG TTTG	0.753	0.690–0.831	0.725	0.638–0.847
Pre-CG CA	0.842	0.723–0.900	0.850	0.782–0.918
Pre-CG PTA	0.863	0.756–0.938	0.786	0.783–0.891
Pre-CG RPW	0.773	0.696–0.935	0.735	0.640–0.846
Pre-CG RPT	0.812	0.784–0.942	0.750	0.582–0.900
Pre-CG WA	0.823	0.753–0.931	0.789	0.623–0.881
Pre-CG MWI	0.830	0.693–0.906	0.738	0.527–0.864
Pre-CG LPFA	0.964	0.953–0.974	0.957	0.920–0.976
Post-CG TTTG	0.806	0.734–0.905	0.730	0.668–0.836
Post-CG CA	0.860	0.735–0.922	0.813	0.756–0.943
Post-CG PTA	0.835	0.751–0.934	0.814	0.729–0.898
Post-CG RPW	0.830	0.759–0.965	0.820	0.779–0.926
Post-CG RPT	0.848	0.738–0.942	0.819	0.758–0.940
Post-CG WA	0.820	0.691–0.834	0.784	0.624–0.890
Post-CG MWI	0.916	0.857–0.954	0.903	0.816–0.942
Post-CG LPFA	0.958	0.926–0.985	0.957	0.918–0.973

*ICC* intra-class correlation coefficient, *SG* study group, *TTTG* tibial tuberosity–trochlear groove distance, *CA* congruence angle, *PTA* patellar tilt angle, *RPW* relative patellar width, *RPT* relative patellar width, *WA* Wiberg angle, *MWI* modified Wiberg index, *LPFA* lateral patellar facet angle, *CG* control group

### Evaluation indexes of alignment of the patello-femoral joint and knee function

Preoperatively, the data regarding alignment of the patello-femoral joint were not significantly different between the two groups (tibial tuberosity–trochlear groove distance (TT–TG), *P* = 0.543; congruence angle, *P* = 0.586; patellar tilt angle, *P* = 0.763; Kujala score, *P* = 0.554) (Table 3). At the last follow-up, the TT–TG showed significant difference (SG, 11.63 mm; CG, 15.26 mm; *P* < 0.001) between both groups. The congruence angle showed highly significant differences between the groups (SG, 9.55°; CG, 23.26°; *P* < 0.001). Significant differences were seen in well-known measurements such as patellar tilt angle (SG, 8.20°; CG, 14.86°; *P* < 0.001). Greater significances were found in the Kujala score: 89.60 in the SG versus 68.65 in the CG (*P* < 0.001) (Table 4). In addition, 20 patients in CG were diagnosed as recurrent patellar dislocation. However, for the apprehension test, there were 4 patients experiencing the patellar lateral shift that exceeded 1.5 cm in SG, suggesting significant difference between both groups. The Crosby and Insall grading system revealed 19 (54%) excellent results in SG Versus 0 (0%) in CG, and 0 (0%) patients in SG and 12 (34%) patients in CG reported ‘poor’ results. For all patients, knee function scores improved significantly after surgery.

**Table 3:** Preoperative evaluation of alignment of the patello-femoral joint and knee function

Indexes	SG	CG	*P* value*
Mean TTTG (SD)	15.86 (1.64)	15.58 (1.45)	0.543
Mean CA (SD)	22.68 (3.32)	23.24 (2.79)	0.586
Mean PTA (SD)	14.35 (2.63)	14.68 (2.38)	0.763
Mean Kujala score (SD)	56.58 (2.73)	58.69 (3.28)	0.554

*SG* study group, *CG* control group, *TTTG* tibial tuberosity–trochlear groove distance, *CA* congruence angle, *PTA* patellar tilt angle

*Student’s *t* test

**Table 4:** Follow-up results of alignment of the patello-femoral joint and knee function

Indexes	SG	CG	*P* value*
Mean TTTG, (SD)	11.63 (1.68)	15.26 (1.43)	< 0.001
Mean CA, (SD)	9.55(3.62)	23.26 (2.94)	< 0.001
Mean PTA, (SD)	8.20 (2.23)	14.86 (2.86)	< 0.001
Mean Kujala score, (SD)	89.60 (3.58)	68.65 (3.81)	< 0.001
Apprehension sign, n (%)			
< 1.5 cm	31/35 (89)	3/35 (0)	
> 1.5 cm	4/35 (11)	32/35 (91)	
Crosby and Insall grading system, n (%)			
Excellent	19/35 (54)	0/35 (0)	
Good	12/35 (34)	3/35 (9)	
Fair	4/35 (12)	20/35 (57)	
Poor	0/35 (0)	12/35 (34)	

*SG* study group, *CG* control group, *TTTG* tibial tuberosity–trochlear groove distance, *CA* congruence angle, *PTA* patellar tilt angle

*TTTG, CA, PTA and Kujala score were calculated using student’s *t* test. The apprehension sign and Crosby and Insall grading system were compared by Pearson’s chi-squared test

### Evaluation indexes of patellar morphology

Preoperatively, the data regarding morphological characteristics of the patella were not significantly different between the two groups (relative patellar width, *P* = 0.184; relative patellar thickness, *P* = 0.738; Wiberg angle, *P* = 0.874; modified Wiberg index, *P* = 0.076; lateral patellar facet angle, *P* = 0.385) (Table 5). At the last follow-up, the relative patellar width showed no significant difference (SG, 54.61%; CG, 52.87%; *P* = 0.086) between both groups. The relative thickness was not significantly different between the groups (26.07% in the SG vs. 25.02% in the CG) (*P* = 0.243). In contrast, the mean Wiberg angle showed highly significant differences between the groups (SG, 136.25°; CG, 122.65°; *P* < 0.001). Significant differences were seen in well-known measurements such as modified Wiberg index (SG, 1.23; CG, 2.65; *P* < 0.001). The mean lateral patellar facet angle showed the greater difference: 23.35° in the SG versus 15.26° in the CG (*P* < 0.001) (Table 6).

**Table 5:** Preoperative evaluation of patella morphological characteristics

Indexes	SG	CG	*P* value*
Mean RPW (SD)	46.67 (7.64)	44.85 (6.85)	0.184
Mean RPT (SD)	17.75 (4.30)	17.24 (4.78)	0.738
Mean WA (SD)	165.35 (8.71)	164.65 (8.35)	0.874
Mean MWI (SD)	1.56 (0.73)	1.32 (0.76)	0.076
Mean LPFA (SD)	21.65 (2.31)	22.85 (2.15)	0.385

*SG* study group, *CG* control group, *RPW* relative patellar width, *RPT* relative patellar width, *WA* Wiberg angle, *MWI* modified Wiberg index, *LPFA* lateral patellar facet angle

*Student’s *t* test

**Table 6:** Follow-up results of morphological characteristics of the patella

Indexes	SG	CG	*P* value*
Mean RPW (SD)	54.16 (2.76)	52.87 (3.31)	0.086
Mean RPT (SD)	26.07 (2.05)	25.02 (3.28)	0.243
Mean WA (SD)	136.25 (7.76)	122.65 (7.31)	< 0.001
Mean MWI (SD)	1.23 (0.18)	2.65 (0.45)	< 0.001
Mean LPFA (SD)	23.35 (3.41)	15.26 (3.66)	< 0.001

*Student’s *t* test

SG, study group; CG, control group; RPW, relative patellar width; RPT, relative patellar width; WA, Wiberg angle; MWI, modified Wiberg index; LPFA, lateral patellar facet angle

## Discussion

The key finding of the current study was that the patellar morphology could be improved by early surgical correction in children with the setting of recurrent patellar dislocation compared with conservative management. The findings of this study are of important reference value in clinical practice for the surgical treatment of recurrent patellar dislocation, as the improvement of patella and femoral trochlear in patients of patellar dislocation may allow for the avoidance of an additional surgical procedure.

However, the surgical outcome of isolated medial patello-femoral ligament reconstruction for recurrent patello-femoral instability in adults was reliable [34]. In children, soft-tissue surgery seems to be the only treatment option, as the tibial and femoral physes may be injured by bony procedures, possibly leading to premature closure [15, 35–37]. To resolve this issue, Wang et al. [9] reported the MPR plasty, in which the MPR was pulled proximally and laterally to the medial margin of the patella to shorten the MPR, which had a strong transverse tension in quiescent conditions. The VMO was pulled laterally and distally to the medial edge of the patella to restore the superior-oblique bundle of the MPR. In addition, the overlapped tissues were sutured together, which further restored the ‘meshing’ structure of the MPR. In previous studies [35, 38, 39], several types of soft-tissue techniques were reported, such as the medial patellar retinaculum constriction technique, the modified adductor sling technique and the 3-in-1 treatment technique. Compared with the three techniques, MPR plasty has the advantages of being a simple operation, little trauma and few complications. With regard to trochlear dysplasia, the result that femoral trochlear morphology can be improved by early surgical correction in children has been well studied [9], but the patella has not been adequately measured. Fucentese et al. [40] indicated that for patients with trochlear dysplasia, the patellar had a smaller medial facet, and compared with the control group, patella type II and type III had a higher prevalence. Panni et al. [41] found an association between patellar morphology type and femoral trochlear dysplasia grade III as well as a correlation between patella tilt and patella shape. Li et al. [8] reported that patients with trochlear dysplasia had a patella of smaller width, thinner thickness, and more flattened articular facet. Niu et al. [42] studied 40 knees from 20 rabbits that were divided into an experimental group (undergoing a medial soft tissue restraint release) and control group (no surgical interventions). The study demonstrated that the shape and articular surface of the patella became more flattened after patella dislocation in experimental group. However, to our knowledge, no authors have observed the potential effect of the femoral trochlea on the development of the patella in children. The capacity for articular remodeling is well known in children as exemplified in the dysplastic knee [36]. Therefore, we believe that it is of great significance to propose that restoration of more normal biomechanics at the patello-femoral joint may affect the development of the patella. As with the hip, our study suggest that remodeling can occur at the patella and this potential is most apparent in children [43].

Patellar morphology was the focus of the study which yielded meaningful results. The present study showed that the mean Wiberg angle and the mean lateral patellar facet angle were bigger in the knees after the medial patellar retinacular plasty, while the modified Wiberg index was smaller in the knees with medial patellar retinacular plasty, as compared with those with conservative treatments. In the study, the relative patellar width and the relative thickness showed no significant difference between both groups, which was consistent with the results of Otto A [33]. In contrast, the mean Wiberg angle was 136.25° larger in the SG compared with that of conservative treatments (the CG, 122.65°, *P* < 0.001), the modified Wiberg index was 1.23 smaller in the SG compared with that in the CG (2.65, *P* < 0.001), and the mean lateral patellar facet angle reflected a significant difference between the groups (*P* < 0.001). A significant conclusion can be inferred from these results that patellar morphology can be remodeled following early surgical correction of patellar dislocation in children. However, it is of significance to explain this phenomenon and ascertain the transformation of the patellar morphology.

**Fig. 8:**
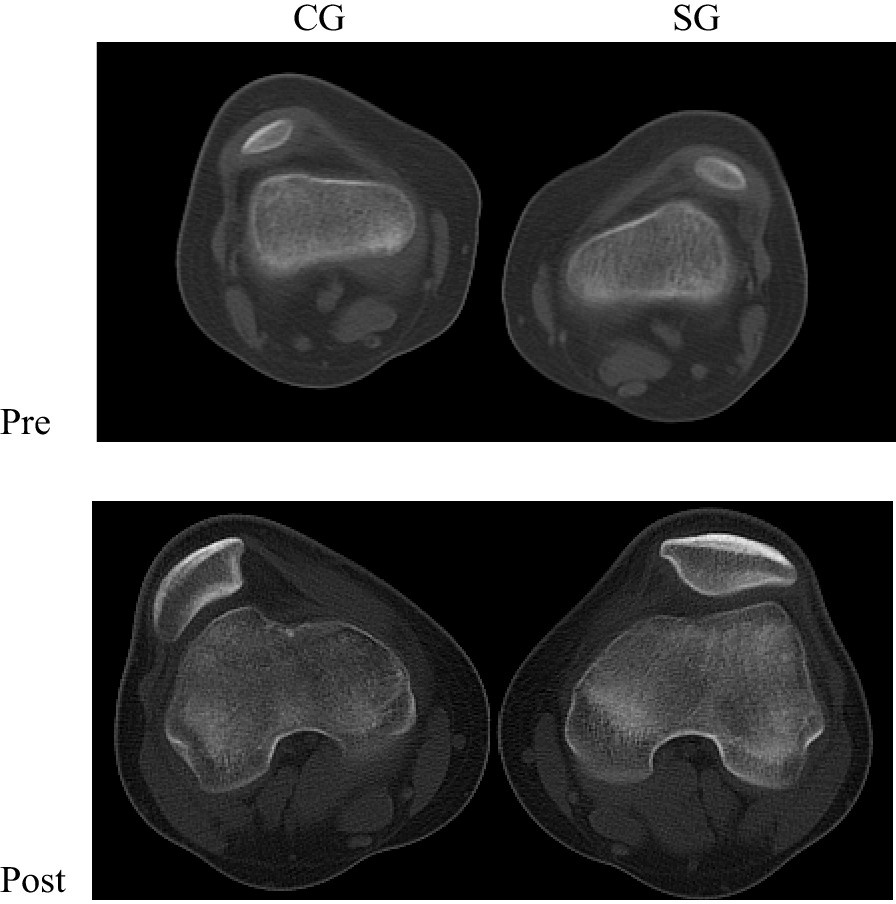
CT scans of patients surgically treated in the study group and conservatively treated in the control group. The scans show remodeling of the patella at the last follow-up in SG patients, which was not seen in CG. CG, control group; SG, study group; Pre, preoperatively; Post, postoperatively

It is acknowledged that the improved congruency of the patello-femoral joint and the mechanical stress of the patella are the basis of its biomechanical function [44]. Stress stimulation plays an important role in bone development [45]. In the patello-femoral joint, stress is transmitted from the articular cartilage to the bone, and because of stimulus transmission, the remodeling has been documented at the femoral trochlear [9]. Furthermore, Fucentese SF et al. [40] reported that a decreased pulling effect of medial patello-femoral ligament resulted in a theoretically shortened medial patellar facet in children. Thus, the patella may be remodeled, when the medial patello-femoral ligament tension returns to normal and the patella rematches the corresponding surface of femoral trochlea. In SG, the patella was remodeled and showed a similar normal morphology at the last follow-up, which was not seen in CG (Fig. 8). The reason might be that the patella was located in the trochlear groove postoperatively.

This study has several limitations. First, CT could be used to describe the osseous structure, rather than revealing the corresponding cartilage surface [46]. Second, due to the small sample size, the results of the study might be different from a larger study. Third, the single transverse sections were used, which might not effectively describe all the morphological changes of the patella. Consequently, the sagittal sections should also be considered in order of precise evaluation [12].

In conclusion, to our knowledge, the present study is the first one to compare patellar morphology in conservative management and surgery in children. The key finding was that compared with conservative management, the patellar morphology could be significantly remodeled to accommodate the patello-femoral joint movement according to early surgical treatment in children with recurrent patellar dislocation.

## Data Availability

The datasets used and/or analyzed during the current study are available from the corresponding author on reasonable request.

## Abbreviations

SG: Study group
CG: Control group
CT: Computed tomography
MPR: Medial patellar retinaculum
MPR: Medial patellar retinaculum
PDS-II: (Polydioxanone) synthetic absorbable suture
SPSS: Statistical Package for the Social Sciences
TTTG: Tibial tuberosity–trochlear groove distance
CA: Congruence angle
PTA: Patellar tilt angle
MLW: Medial–lateral width
PW: Patellar width
PT: Patellar thickness
ICC: Intra-class correlation coefficient
RPW: Relative patellar width
RPT: Relative patellar width
WA: Wiberg angle
MWI: Modified Wiberg index
LPFA: Lateral patellar facet angle
Pre: Preoperatively
Post: Postoperatively
